# Impact of a first psychosis program in functional variables after two years of follow-up

**DOI:** 10.1192/j.eurpsy.2021.1417

**Published:** 2021-08-13

**Authors:** M. Martinez, N. Pereda, E. García De Jalón, T. Lizarbe, A. Aquerreta, F. Monclus, M. Otero

**Affiliations:** Salud Mental. Primeros Episodios, Servicio Navarro de Salud, Pamplona, Spain

**Keywords:** Early-Phase Psychosis, schizophrénia, psychosis, early intervention

## Abstract

**Introduction:**

Early Intervention Services for Early-Phase Psychosis have shown efficacy and effectiveness (Correl C, JAMA). In Pamplona, Spain, there is an Early Intervention Program that has been providing multiprofesional assistance for First Psychotic Patients for the last two years.

**Objectives:**

The aim of this study is to analize the longitudinal effects of the different interventions in several functional variables: GAF, Occupational State, CGI-CogS, QLS, Sofas and WHODAS II applied to 240 patients during two years of follow-up.

**Methods:**

We apply an standard evaluation protocol to every patient at different times: premorbid, initial time and at months 6, 12, 18 and 24. We analyse the data with the SPSS statistical program to see the results in these variables.

**Results:**

The GAF scale shows a decline during the first 6 months, but tends to reach and maintain the premorbid levels after a year of treatment. Regarding baseline, patients with normalized jobs or studies are 60.7%. This percentage persists during the next months of follow-up but decline at the 24^th^ month Both the Whodas and Sofas scale show improvement tends. The QLS results show a progresional improvement in every subcale during the whole time of follow-up.

**Conclusions:**

The Early Intervention Services in Psychosis improve, not only psychopatological dimension but also functional areas, what is important for the whole recovery of First Psychotic Patients.

